# The mindful trajectory: Developmental changes in mentalizing throughout adolescence and young adulthood

**DOI:** 10.1371/journal.pone.0286500

**Published:** 2023-06-21

**Authors:** Alex Desatnik, Annie Bird, Avi Shmueli, Ilya Venger, Peter Fonagy

**Affiliations:** 1 Research Department of Clinical, Educational and Health Psychology, University College London, London, United Kingdom; 2 Anna Freud National Centre for Children and Families, London, United Kingdom; 3 Open Door Young People Service, London, United Kingdom; 4 Department of Psychology, Royal Holloway, University of London, Surrey, London, United Kingdom; 5 Weizmann Institute of Science, Rehovot, Israel; 6 Microsoft Israel Development Centre, Herzliya, Israel; University of Padova, ITALY

## Abstract

**Background:**

Mentalizing and psychological mindedness are two key, partially overlapping facets of social cognition. While mentalizing refers to the ability to reflect on one’s own mental states and the mental states of others, psychological mindedness describes the ability for self-reflection and the inclination to communicate with others about one’s own mental states.

**Purpose:**

This study examined the development of mentalizing and psychological mindedness throughout adolescence and into young adulthood, and the interplay between the two with gender and the Big Five Personality Traits.

**Methods:**

432 adolescents and young adults (ages 14–30) were recruited from two independent schools and two universities. Participants completed a set of self-report measures.

**Results:**

A curvilinear trend in both mentalizing and psychological mindedness indicated a gradual development of these capacities with age, peaking in young adulthood.

Across all age groups, females had consistently higher mentalizing scores than males. For females, scores only changed significantly between age bands 17–18 to 20+ (p<0.001), ES (d = 1.07, 95% CI [.1.52–.62]). However, for males, a significant change in scores appeared between two age bands of 14 to 15–16 (p<0.003), ES (d = .45, 95% CI [.82–.07]), and 17–18 to 20+ (p<0.001), ES (d = .6, 95% CI [.1.08–.1]).

The change in psychological mindedness scores differed, and females did not have consistently higher scores than males. Females’ scores were only significantly higher for ages 14 (p<0.01), ES (d = .43, 95% CI [.82–.04]), and 15–16 (p<0.01), ES (d = .5, 95% CI [.87–.11]). As with the development of mentalizing abilities, female scores in psychological mindedness remained stable from 14 to 18 years of age, with a significant change between age bands 17–18 and 20+ (p<0.01), ES (d = 1.2, 95% CI [1.7–.67]). Contrastingly, for males significant change occurred between 15–16, 17–18 (p<0.01), ES (d = .65, 95% CI [1.1–.18]) and 20+ (p<0.01), ES (d = .84, 95% CI [1.5–.2]).

A significant positive association was found between mentalizing and psychological mindedness and the personality traits of Agreeableness, Openness to Experience and Conscientiousness (p<0.0001). Psychological mindedness had a weaker positive correlation with Extraversion and Openness to Experience (p<0.05).

**Conclusions:**

The discussion is focused on the interpretation of the findings in light of social cognition and brain development research.

## Introduction

The development of social cognition has long been a topic of significant interest [1]. Social cognition is a broad term referring to a set of cognitive processes underlying human interaction and understanding of others [2], or simply cognitive processes involving other people [3]. In recent years, much research has focused upon the development of social cognition during adolescence. It has been established that the human brain undergoes a number of important changes during this period—especially in the ‘social brain’ regions associated with social cognition—enabling an individual to become more aware of his/her own mental states and recognise and evaluate the mental states of others [4]. In light of this research, a renewed interest in the development of various facets of social cognition during adolescence has emerged [5,6] with many studies utilising advances in neuroscientific techniques [7–11].

One of the key constructs capturing multiple facets of social cognition is mentalizing [12] Mentalizing defined as “the mental process by which an individual implicitly and explicitly interprets the action of themself and others as meaningful on the basis of intentional mental states such as personal desires, needs, feelings, beliefs and reasons” [13]. There is also a distinction between explicit and implicit mentalizing. Explicit mentalizing refers to ‘thinking and talking about mental states’ [14] and implies a conscious understanding of one’s own mental state and the mental state of others. Implicit mentalizing, also occurring both in relation to self and others, is intuitive and non-conscious like mirroring, turn taking in conversation, and so on.

In the past decade many studies have measured mentalizing in adolescence in relation to attachment and the implications for disorders such as anorexia nervosa and borderline personality disorder, and their treatment [15,16]. However, less focus has been given to the typical development of mentalizing capacity during this period and its association with other aspects of personality and social cognition.

### Development of social cognition in adolescence

The human ability to interact socially necessitates a rapid development of social cognition in early life, moving from simple social skills, like attracting the attention of others [17], to a relatively well developed ability to understand one’s own emotions, the emotions of others and to moderate actions accordingly [18,19]. The mapping of social cognitive development in adolescence is more complex as social demands on the developing individual increase rapidly through this turbulent period. These challenges are especially evident in areas such as peer relationships, reaction to peer pressure, and risk taking. All of these require robust emotion regulation and involve multiple social-cognitive demands such as an ability to identify the impact of others on one’s emotions as well as the capacity to take another’s perspective [20–23]. Social preferences of adolescents also undergo significant change, with the importance of the peer group gradually increasing as time spent around family decreases [24,25]. To match these developmental changes and challenges, a key task of the adolescents cognitive development is to establish a self-regulating and self-aware mind, through the development of executive functioning and social cognition [13]. This developmental picture is further complicated by the recent understanding that brain maturation processes (such as synaptic pruning and myelination and the maturation of white matter) which support higher level emotional and executive functioning, are not invariably linked to puberty and restricted to early adolescence, but go on well into the late teens and early 20s [26]. As a result, adolescence can be understood as an extended period of development, merging into early adulthood.

### Social cognition and gender

Gender differences in social cognitive abilities exist across all age groups. From 6 years old girls are more able to make inferences about the mental states of others, performing better in theory of mind tasks than boys [27] and these differences continue into young adulthood [28]. In adolescence the reasons for women’s superior social cognitive skills are thought to be both biological and social. Biologically, women undergo puberty at an earlier age than men, resulting in faster brain maturation [29]. Socially, the gender intensification theory suggests that just prior to adolescence, gender differences between males and females increase so that each sex conforms more to their traditional gender roles [30], resulting in a further increase in gender differences in social cognitive skills [31].

### Mentalizing and development

Mentalizing is thought to emerge in infancy in the context of early attachment relationships, specifically through the experience of one’s complex emotions contingently and markedly “mirrored” by attachment figures [32]. Mentalizing is thought to develop and operate across four dimensions: 1) automatic—controlled (implicit versus explicit); 2) internally focused—externally focused (inferring mental states based on taking the other’s perspective versus inferring through external behavioural cues), self—other (focus on own mind versus focus on the mind of the other), and cognitive—affective (relying on cognition versus relying on emotion and empathy). Therefore, the ability to mentalize includes a wide range of social cognitive processes concerning mental states such as perception, recognition, description, etc [13,33].

The rapid changes in social demands during adolescence, such as shifting to more complex social environments and developing new close relationships with peers, make mentalizing one of the key facets of social cognition allowing adolescents to successfully meet their developmental challenges [34]. It is suggested that during adolescence, mentalizing develops as rapidly as other facets of social cognition [35]. Acquisition of new cognitive capacities allows for a more complex understanding of how changes in mental states are expressed. Rather than attributing relatively basic emotional states to him/herself and others, the adolescent begins to consider more complex scripts of mental states, emotions and behaviors. However, as these newly acquired capacities are still unstable, they can revert under stress and in the face of ambiguity [36]. While encompassing crucial skills for social functioning and overall wellbeing, mentalizing capacity does not reach its peak until early adulthood.

A number of neuroimaging studies have shown a significant difference in the activation of the mentalizing system between adults and adolescents in tasks requiring an ability to understand the self and others [11]. Furthermore it was shown that theory of mind and executive functions, both strongly implicated in mentalizing, also continue to develop into late adolescence and early adulthood [25,37].

Overall, it appears that various cognitive systems mature at different rates in the developing brain and often gaps between cognition and behavior occur, making adolescence a particularly turbulent period of development [38,39].

In light of this evidence, it is of interest to examine whether other related facets of social cognition show similar patterns of development through adolescence.

### The concept of mentalizing and other related constructs

The concept of mentalizing encompasses multiple phenomena of social cognition, hence there are several constructs that are considered to have a conceptual overlap with the term. These include mindblindness [40], emotional intelligence [41], mindfulness, empathy, and psychological mindedness [42] amongst others. In the present study, we were particularly interested in the overlap with psychological mindedness—a concept which also encompasses a breadth of social cognitive aspects.

Psychological mindedness is defined as “a person’s ability to see relationships among thoughts, feelings, and actions, with the goal of learning the meanings and causes of his experiences and behaviour” [43]. There are several areas of overlap between psychological mindedness and mentalizing: in both concepts the affective and cognitive aspects are equally represented [41,44]. Furthermore, research also indicates that psychological mindedness, like mentalizing, is positively linked with attachment: securely attached and well-adjusted individuals are more likely to exhibit higher levels of psychological mindedness [45]. However, as mentioned previously, mentalizing operates both implicitly and explicitly, whilst psychological mindedness is more concerned with explicit and conscious understanding of mental states, giving more emphasis to the self and one’s own mental states. Furthermore, psychological mindedness captures one’s willingness to talk about one’s own mental states, and as such was found to be a predictor of engagement and outcome in psychotherapy [46,47].

In this study, we were particularly interested in psychological mindedness due to its significant overlap with mentalizing but also the clear distinction between the two. While stemming from a similar theoretical origin and emphasizing both the cognitive and affective aspects of social cognition psychological mindedness focuses on the conscious, explicit and deliberate aspects of thinking and *talking* about internal experiences, interest and inclination to such thinking. We expect their conceptual proximity alongside the clear distinction between the two may allow to better identify the developmental differences between the constructs.

### The big five personality traits and social cognition

Personality affects how individuals interact with the world around them and is an important factor in the development of a wide range of interpersonal and cognitive skills, including social cognition [48]. Personality factors are generally thought to develop relatively early in life and remain stable through adolescence and young adulthood [49,50].

Research into the relationship between personality and the various facets of social cognition is limited and to a large extent focused upon empathy [51,52]. There is limited evidence describing the way the Big Five Personality Traits (Extraversion, Agreeableness, Conscientiousness, Emotional Stability and Openness to Experiences [53]) are associated with social cognition. Another study showed that Openness to Experience and Extraversion were good predictors of psychological mindedness [54]. A positive correlation was shown between the ability to mentalize others and Agreeableness, in particular the compassion facet, as high levels of Agreeableness rely upon accurate interpretation of other people’s mental states followed by appropriate behaviors [55]. None of the previous studies reported the associations between all the big five personality traits and mentalizing, nor have they used self-report to measure both concepts. Therefore, less is known about the associations between the other personality traits and the similarities and differences in their associations with mentalizing and psychological mindedness.

### Aims

The aim of this study was to examine the development of two aspects of social cognition; mentalizing and psychological mindedness, measured through self-report, between early adolescence and young adulthood (14–30) as well as the impact of gender on these changes. Additionally, we set out to explore the previously unestablished associations between the two constructs and the Big Five Personality Traits.

It was hypothesized that both mentalizing and psychological mindedness would continue developing similarly through adolescence and into young adulthood due to the significant conceptual overlap between the two. It was of interest to explore whether there were differences in the development of these two facets of social cognition that would account for the differences between these two capacities. It was also hypothesized that there would not be a significant shift in personality associated with age.

Finally, we intended to explore whether the finding of this self-report study would be broadly in line with the evidence from neuroscientific and experimental literature to evaluate whether the use of self-report is a feasible measure of developmental changes in social cognition during adolescence.

## Method

### Participants

A total of 432 adolescents and young adults aged between 14–30 participated in the study (M age = 17.14 years, SD = 4.2). The participants were divided into four groups representing developmental age periods: 14 years old (N = 131, 64 males, 67 females), 15–16 years old (N = 141, 60 males, 81 females), 17–18 years old (N = 72, 35 males, 37 females), 20–30 years old (N = 89, 38 males, 51 females; (M age = 24.85 years, SD = 2.4)). 82% indicated their mother tongue as English, 8% other European, 8% other. No other demographic information was collected. Participants were recruited from two independent single sex schools and two universities in London, United Kingdom. The university students were recruited from seminar groups: 38% of the participants studied humanities, 33% psychology and 29% sciences. The study was approved by the University College London (UCL) Ethics Committee (0384/033).

Participants over the age of 16 were asked to sign a consent form and informed that they were free to withdraw or refuse to participate in the study at any point. For participants under the age of 16 the schools used a passive permission procedure asking parents to sign if they didn’t wish their children to participate in the study.

### Measures

#### The reflective functioning questionnaire (RFQ)

The term Reflective Functioning has been used as an operationalization of the mentalizing concept [56]: it is often used synonymously with mentalizing [57]. The original RFQ [58] was developed to measure deficits in mentalizing by measuring the level of certainty and uncertainty about self and others’ mental states on two separate scales. Mentalizing is characterized by a recognition of the opaqueness of mental states and therefore requires a degree of modesty about knowing one’s own and others’ mind. Therefore, both an excessive certainty and uncertainty indicate difficulties in genuine mentalizing [58]. Several studies had used the RFQ and its adaptations with an adolescent sample [57,59]. The version of the RFQ used in the present study consists of 15 of the original items coded to provide a single scale, measuring strength or weakness in mentalizing, and is considered more suitable for assessing mentalizing in non-clinical samples [64]. Each item requires the participant to provide a response on a Likert scale ranging from 1 (strongly disagree) to 6 (strongly agree), depending upon the extent to which they felt the item pertained to them. For 10 items strong agreement represents high level of mentalizing (e.g.: “I believe that people can see a situation very differently based on their own beliefs and experiences”), and for five items strong agreement represents low level of mentalizing (e.g.: “I get confused when people talk about their feelings”). The higher total coded score indicates better mentalizing. The internal consistency of the scale was shown to be high (α = 0.78) and the test–retest reliability of the measure over a period of 3 weeks was excellent (r = 0.84 p<0.001) [58].

#### The psychological mindedness scale (PMS)

Developed by Conte and colleagues [54] this 45 item self-report measure presents possible responses from 1 (strongly agree) to 4 (strongly disagree). For 20 items, response 1 indicates high psychological mindedness (e.g.: “I’ve found that when I talk about problems to someone else, I come up with ways to solve them that I hadn’t thought of before”), for the remaining 25 response 4 indicates high psychological mindedness (e.g.: “I never found that talking to other people about my worries helps much”). The higher total score in this measure indicates higher levels of psychological mindedness. The scale is widely used in both research and clinical practice with an established validity. The temporal stability of the scale has been assessed over two weeks (r = 0.92) [54] and the internal consistency of the scale has also been shown to be high (α = 0.87) [60].

#### The ten item personality inventory (TIPI)

Developed by Gosling and colleagues [61], this is a short instrument consisting of 10 items with suggested responses on the Likert scale from 1–7 designed to assess the Big Five Personality Traits: Extraversion, Agreeableness, Conscientiousness, Emotional Stability and Openness to Experiences. This brief questionnaire demonstrated high correlation (mean r = 0.77) with the original 50 item measure [61]. The original measure was found suitable to assess adolescents aged 13 and over [62] and showed gender invariance on all the personality dimensions, except for Agreeableness, which was found to be higher in females [57].

### Procedure

The adolescent participants were invited to fill in the questionnaire pack in pencil-paper format in the classroom before or during their school day. Researchers and the teacher were present in the classrooms while the questionnaires were filled in. The completed questionnaires were then collected by the researcher. Half of the young adult group filled in the questionnaire pack in pencil-paper format returning the competed forms to the researchers, while the other half completed the questionnaire pack on-line. It was explained to all the participants that their responses were fully anonymous and that they were free to withdraw from the study at any point. There were 12 participants in the adolescent groups and 2 in the young adult group who refused to take part in the study. The acquired data was coded and analyzed using the SPSS 27.0 software package.

## Results

Table 1 presents the intercorrelations between the RFQ, PMS, the five TIPI scales, age and gender (See Table 2 for distribution information for the measures used in the study). These correlations indicate that, as predicted, the RFQ had a strong and significant positive correlation with PMS, showing the convergent validity of both measures. Both the RFQ and the PMS were significantly correlated with gender, age and three of the five personality traits—Agreeableness, Conscientiousness and Openness to Experiences. Furthermore, the PMS also had a weaker correlation with Extraversion and Emotional Stability.

**Table 1:** Intercorrelations among examined variables.

	**RFQ**	**PMS**	**Gender**	**Age**	**Extraversion**	**Agreeable ness**	**Conscientiousness**	**Emotional Stability**	**Openness to Experiences**
**RFQ**	−								
**PMS**	.668 (***)	−							
**Gender**	.302 (***)	.184 (***)	−						
**Age**	.401 (***)	.461 (***)	.091	−					
**Extraversion**	.051	.128 (*)	.049	− .105 (*)	−				
**Agreeableness**	.380 (***)	.346 (***)	.188 (***)	.140 (**)	.039	−			
**Conscientiousness**	.302 (***)	.272 (***)	.039	.128 (*)	− .010	.294 (***)	−		
**Emotional Stability**	.072	.137 (*)	− .155 (**)	.007	.152 (**)	.207 (***)	.225 (***)	−	
**Openness to Experiences**	.308 (***)	.284 (***)	.065	.065	.267 (***)	.175 (***)	.031	.055	−

*p<0.05,

**p<0.01,

***p<0.0001 (2 tailed).

**Table 2:** Mean, standard deviations, skewness and kurtosis statistics for the study measures.

	Mean	Std. Deviation	Skewness	Std. Error	Kurtosis	Std. Error
**RFQ**	4.299	.471	-.488	.120	.977	.240
**PMS**	125.685	13.540	-.140	.135	.545	.269
**Extraversion**	9.618	2.751	-.439	.127	-.274	.254
**Agreeableness**	9.509	2.138	-.157	.128	.068	.255
**Conscientiousness**	9.774	2.878	-.612	.127	-.054	.254
**Emotional Stability**	8.819	2.909	-.233	.128	-.659	.254
**Openness to Experiences**	10.844	2.015	-.600	.127	-.041	.254

### Age and gender effects

#### Mentalizing

To assess the developmental progression of the scores and the moderating effects of gender a series of two tailed t-tests was conducted.

**Fig 1:**
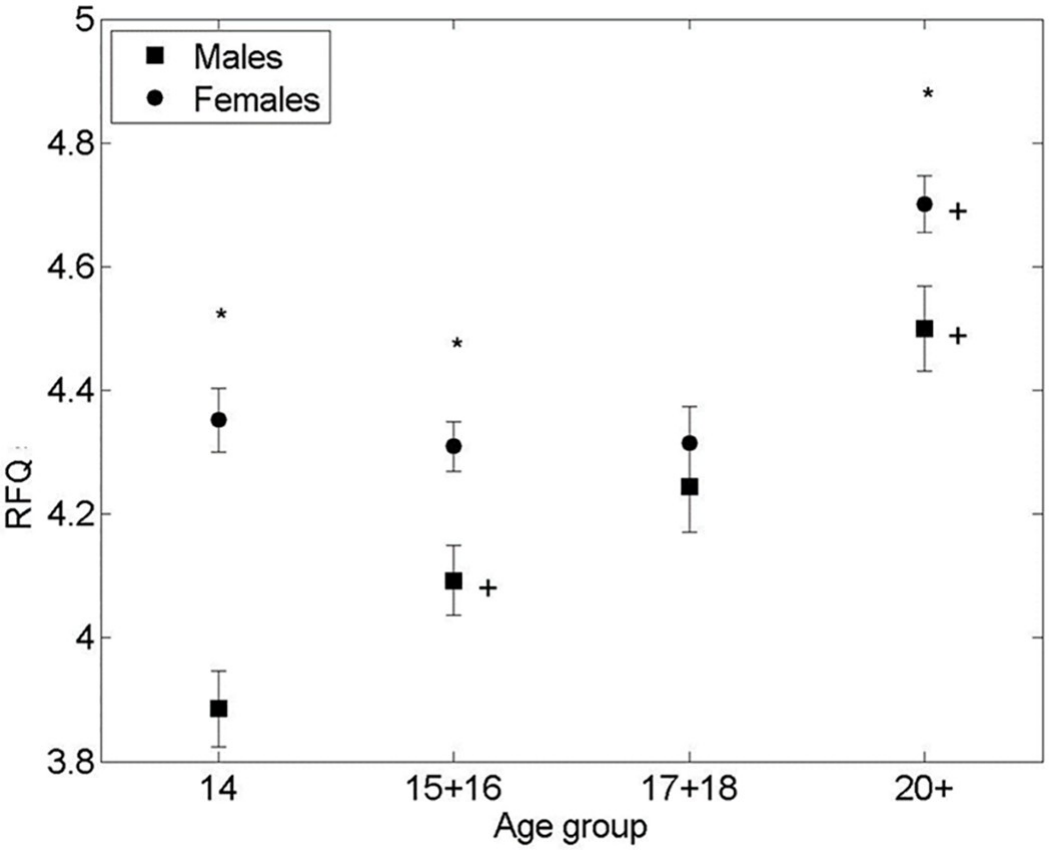
Mean RFQ score vs. age group of respondents. * significant difference between the means of males and females in an age group (2-sided Student’s T-test p-value < 0.001). + significant difference between the means of males/females between adjacent age groups (T-test p-value < 0.01). Bars are standard error of the mean.

As shown on Fig 1, there were significant differences in the RFQ score between males and females in age groups 14 (p<0.001), ES (d = 1.02, 95% CI [1.41–.63]) and 15–16 (p<0.001), *ES (d =* .*55*, *95% CI [*.*91–*.*19])*, with females scoring higher than males. The difference seemed to dissipate for age group 17–18 to then appear again at 20+ (p<0.001), *ES (d =* .*52*, *95% CI [*.*94–*.*08])*. A significant increase in RFQ score has occurred in males from the age group 14 to age group 15–16 (p<0.003), *ES (d =* .*45*, *95% CI [*.*82–*.*07])*. No significant change was observed between age group 15–16 and 17–18 but another significant increase occurred in males between the ages of 17–18 to 20+ (p<0.01). The RFQ scores were stable in females aged 14 to 18 showing no significant change, but similarly to males a significant increase occurred between the ages of 17–18 to 20+ (p<0.001), ES (d = .6, 95% CI [.1.08–.1]). Overall, the females had a consistently higher RFQ score than males.

#### Psychological mindedness

**Fig 2:**
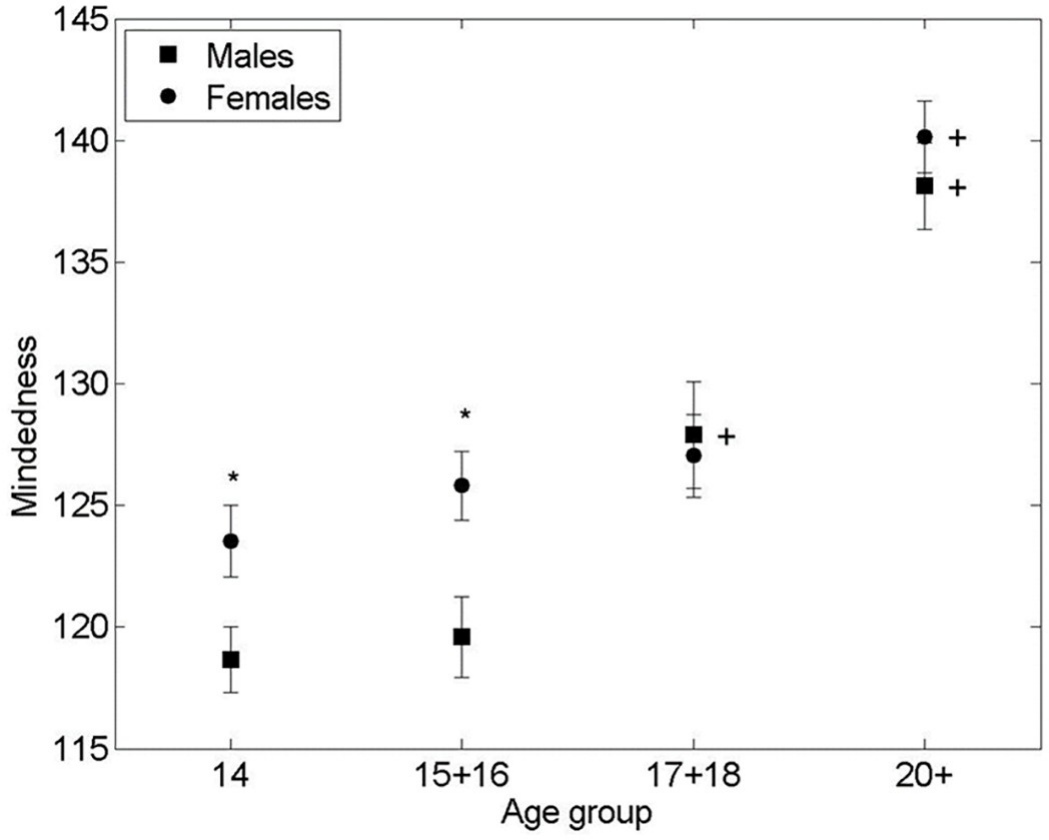
Mean mindedness score vs. age group of respondents. * significant difference between the means of males and females in an age group (2-sided Student’s T-test p-value < 0.001). + significant difference between the means of males/females between adjacent age groups (T-test p-value < 0.01). Bars are standard error of the mean.

As evident from Fig 2 the change in PMS scores also showed an interesting dynamic. As with the RFQ, significant differences in PMS scores were found between males and females in age groups 14 (p<0.01), ES (d = .43, 95% CI [.82–.04]) and 15–16 (p<0.01), *ES (d =* .*5*, *95% CI [*.*87–*.*11])*, with females scoring higher than males. There were no significant gender differences found in the 17–18 age group nor in the 20+ group. A significant increase in PMS score was observed in males between the ages group of 15–16 and 17–18 (p<0.01), ES (d = .65, 95% CI [1.1–.18]) and again from 17–18 to 20+ (p<0.01), ES (d = .84, 95% CI [1.5–.2]). While similarly to the RFQ the scores of females seemed stable between the ages of 14 to 18, a significant shift in PMS scores occurred between the age of 17–18 and 20+ (p<0.01), ES (d = 1.2, 95% CI [1.7–.67]) (see Fig 2).

#### The big five personality traits

**Fig 3:**
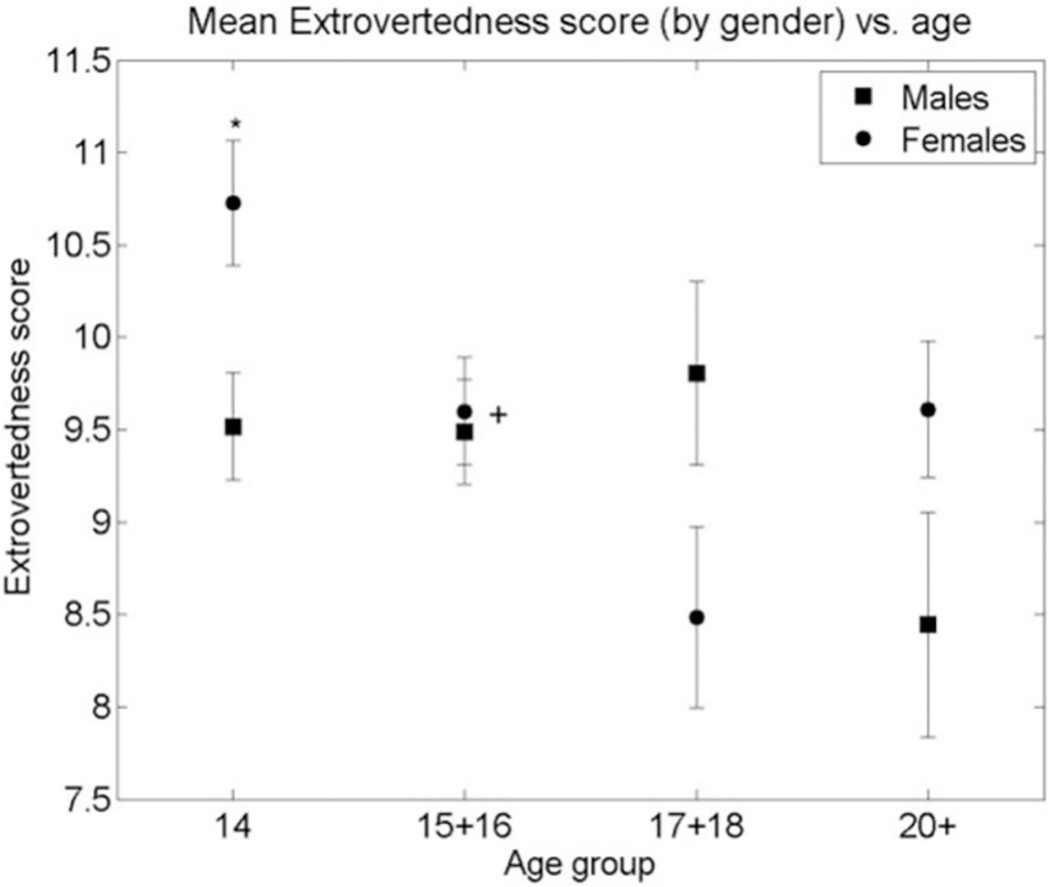
Mean Extraversion score (by gender) vs. age group of respondents. * significant difference between the means of males and females in an age group (2-sided Student’s T-test p-value < 0.001). + significant difference between the means of males/females between adjacent age groups (T-test p-value < 0.01). Bars are standard error of the mean.

**Fig 4:**
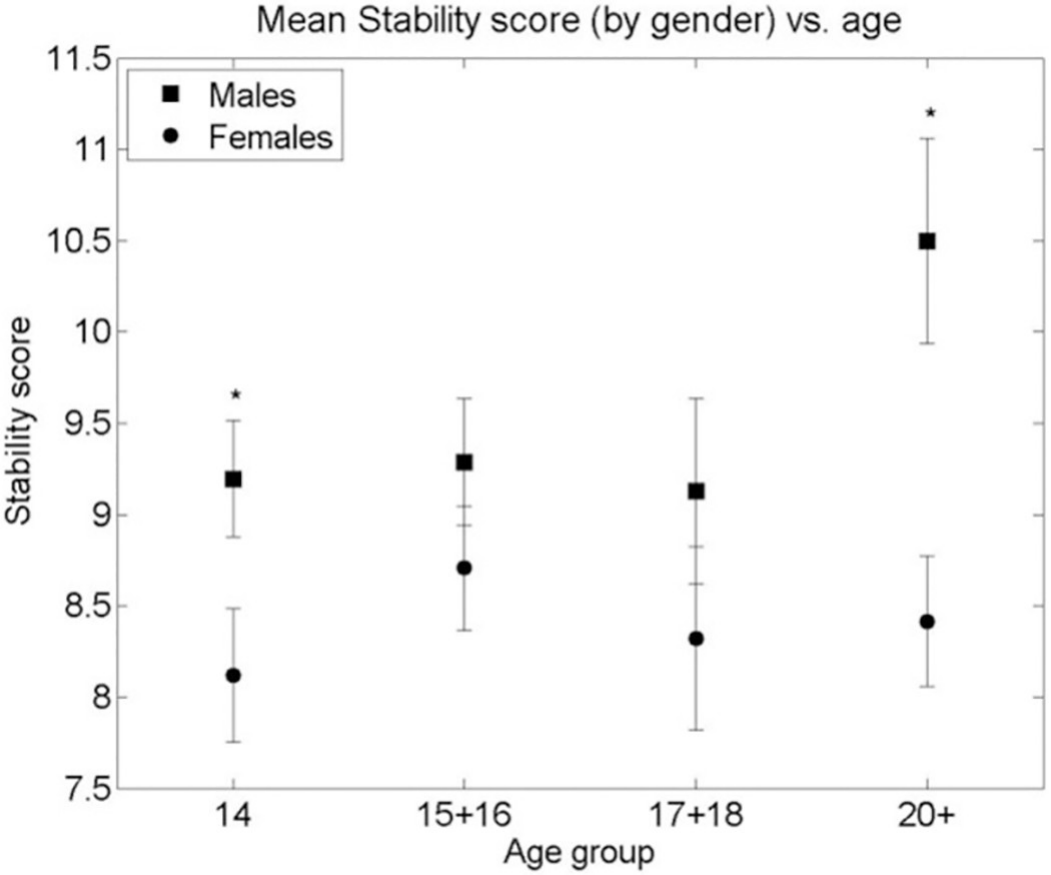
Mean Stability score (by gender) vs. age group of respondents. * significant difference between the means of males and females in an age group (2-sided Student’s T-test p-value < 0.001). + significant difference between the means of males/females between adjacent age groups (T-test p-value < 0.01). Bars are standard error of the mean.

**Fig 5:**
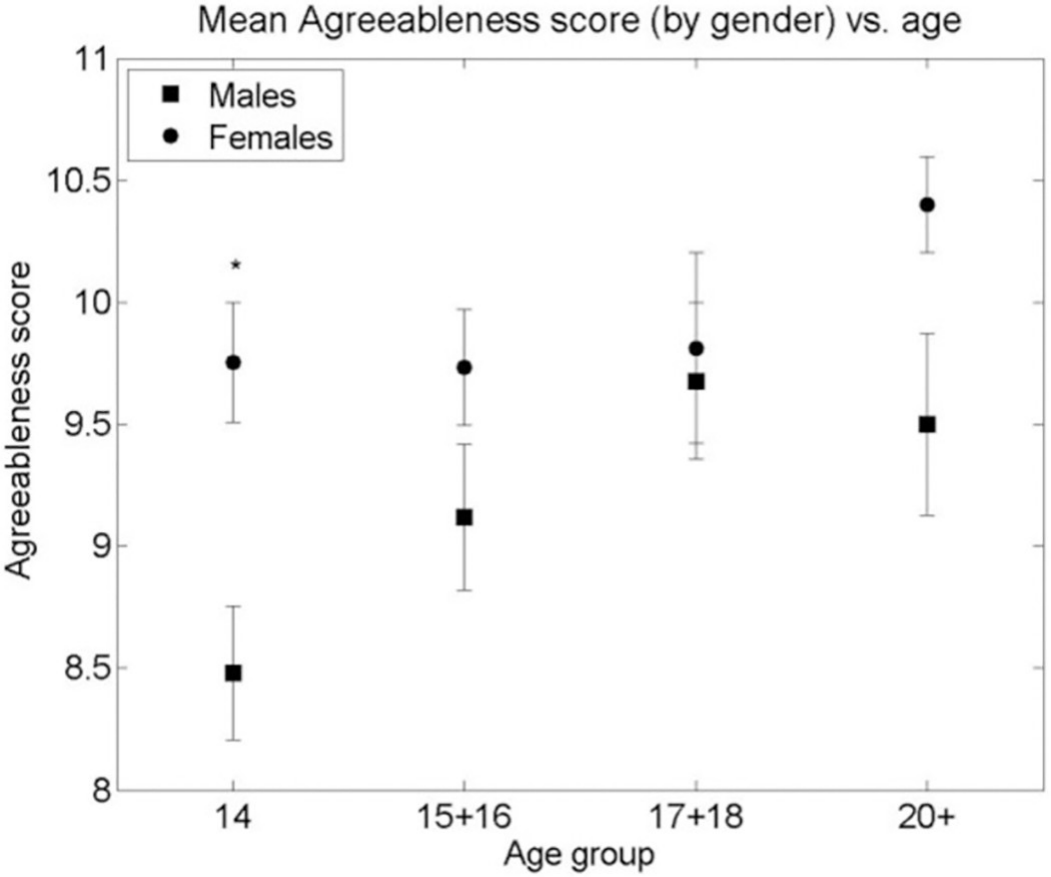
Mean Agreeableness score (by gender) vs. age group of respondents. * significant difference between the means of males and females in an age group (2-sided Student’s T-test p-value < 0.001). + significant difference between the means of males/females between adjacent age groups (T-test p-value < 0.01). Bars are standard error of the mean.

**Fig 6:**
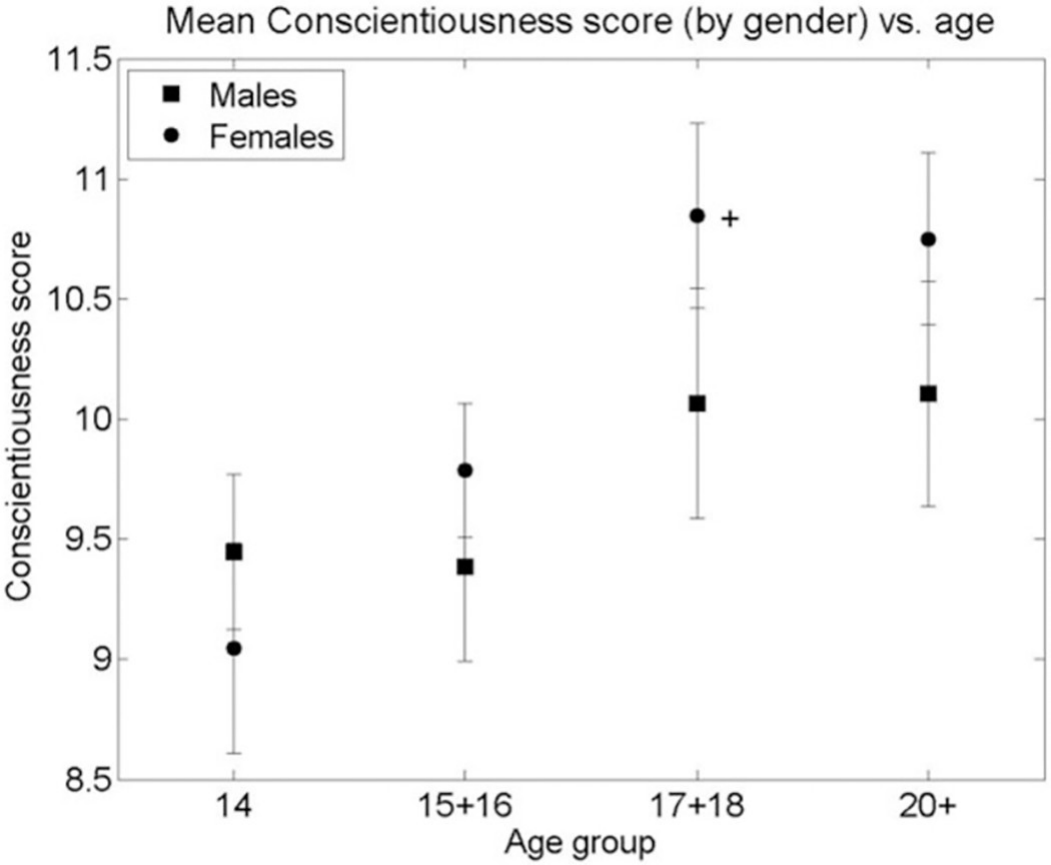
Mean Conscientiousness score (by gender) vs. age group of respondents. * significant difference between the means of males and females in an age group (2-sided Student’s T-test p-value < 0.001). + significant difference between the means of males/females between adjacent age groups (T-test p-value < 0.01). Bars are standard error of the mean.

**Fig 7:**
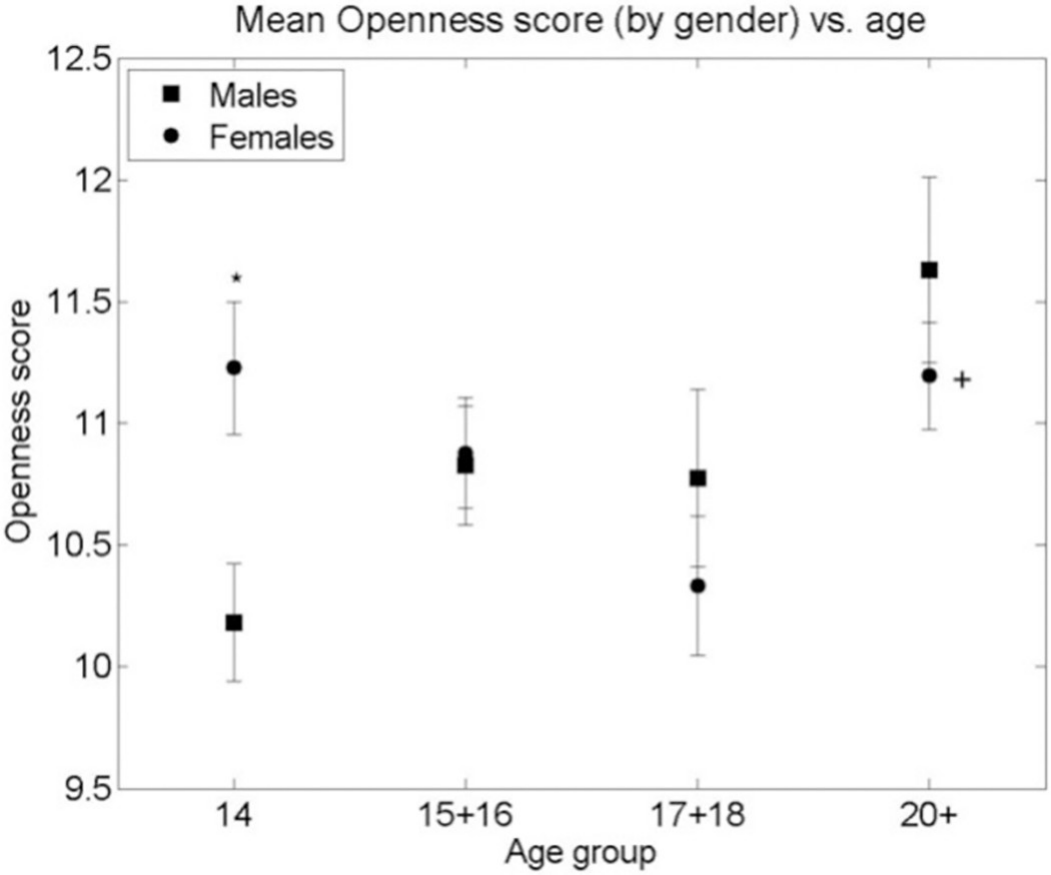
Mean Openness score (by gender) vs. age group of respondents. * significant difference between the means of males and females in an age group (2-sided Student’s T-test p-value < 0.001). + significant difference between the means of males/females between adjacent age groups (T-test p-value < 0.01). Bars are standard error of the mean.

A similar analysis of the Big Five Personality Traits measured by the TIPI showed neither a significant curvilinear relationship with age nor a systematic difference between genders, as can be seen in Figs 3–7. Nevertheless, some differences were found. A significant difference between males and females in Extraversion (*ES (d =* .*47*, *95% CI [*.*83–*.*1])*, Agreeableness *ES (d =* .*61*, *95% CI [*.*98–*.*24])* and Openness to Experiences *ES (d =* .*5*, *95% CI [*.*87–*.*14])* was found at age group 14, all at (p < 0.01), levelling out in all other age groups as the Extraversion scores in females decreased to levels similar to males (p < 0.01), *ES (d =* .*04*, *95% CI [*.*4–*.*31])* and the male scores on Agreeableness and Openness to Experiences increased (though not to statistically significant levels) to those similar in females for the 15–16 age group. The only score showing a different picture is Emotional Stability where a significant difference between males and females is seen in the age group 14 (p < 0.01), *ES (d =* .*39*, *95% CI [*.*02–*.*74])*. This then disappears at age groups 15–16, 17–18 but reappears at the age group of 20+(p < 0.01), *ES (d =* .*7*, *95% CI [*.*11–1*.*28])*. Finally, no significant gender differences were found in the Conscientiousness score, with only an insignificant increase in Conscientiousness in females between the age groups 15–16 and 17–18 (p < 0.05), *ES (d =* .*44*, *95% CI [*.*85–*.*02])*.

## Discussion

The present self-report study sought to establish the developmental changes in mentalizing across different age groups in adolescents and early adulthood and its interplay with psychological mindedness and personality. To the best of our knowledge, this is the first investigation reporting associations between demographic variables (age and gender) and self-reported mentalizing, psychological mindedness and the “big five” personality traits in an adolescent community population. As hypothesized, the results confirmed that mentalizing capacity increased from early to late adolescence into young adulthood, with the general trend apparent in both genders. A similar trend was evident in psychological mindedness. Age and gender differences were found in the development of both these social cognitive attributes. An association of both with personality traits was also established. These findings are discussed in light of social cognition and mentalizing theories below.

### The developmental change

As predicted, mentalizing improved from early to late adolescence and into young adulthood; overall these trends were evident for both genders. This finding is consistent with previous research [35] and can be understood in the context of developments in the prefrontal cortex involved in capacities such as self-awareness and theory of mind or the ability to attribute complex mental states to others [63]. In the process of development adolescents accumulate new social experiences and are able to perceive their social environment in a more complex manner facilitated by these newly acquired capacities.

The results of the present study show a significant increase in mentalizing at around 14 and 15 years, followed by a temporary stagnation, and then followed by a further marked increase between the ages of 17–18 and 20+. The current study used the RFQ which comprises both dimensions of mentalizing, the self and other. A core capacity for mentalizing the other is perspective taking—meaning to “simulate” the thoughts and mental states of others or to ascribe an accurate interpretation and understanding of another person’s motives. This capacity also undergoes a process of significant developmental change with a notable reduction in puberty. It is then gradually regained with substantial improvement noticeable around the ages of 14–16 [64]. These developmental shifts are in line with the assumption that the limited capacity of adolescents to take perspective is related to a burst of proliferation and extensive and less efficient frontal activation. As development continues, the better myelinated brain allows for improved performance and more activation of the frontal cortex [65]. Based on the results of the present study it is possible to suggest that after regaining the capacity for perspective taking and following a period of stagnation this capacity continues to improve when young people are in their 20’s and possibly beyond that time. This view is consistent with research which suggests that the interplay between theory of mind and executive function continues to develop well into late adolescence and even perhaps young adulthood, showing significant improvements in related tasks in groups of adolescents aged 14–17 and young adults aged 19–27 [10]. Future research into the lifespan development of social cognition functions, starting with younger prepubescent age groups could shed more light on this process.

**Fig 8:**
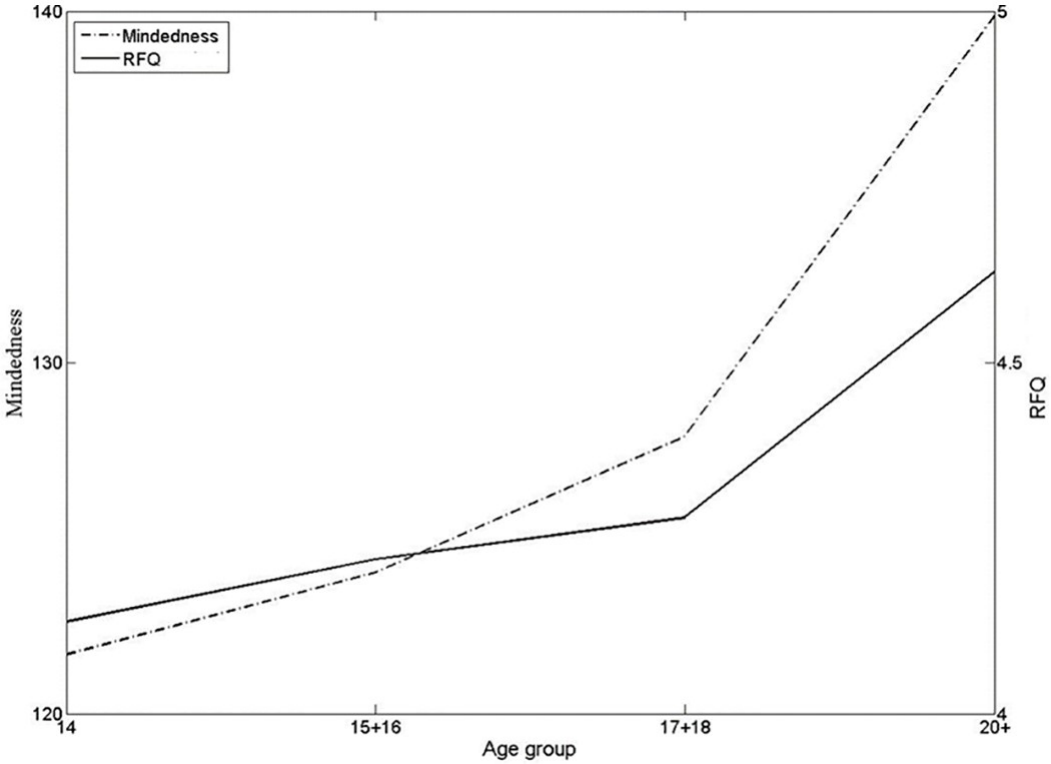
The effect of age upon average RFQ Scale 1 and PMS scores.

As was hypothesized, the developmental patterns appear to be common to several facets of social cognition. Supporting this is the curvilinear pattern of development of psychological mindedness that resembles the pattern of mentalizing development as illustrated in Fig 8.

However, no significant pattern was observed for any of the personality dimensions. Although some minor developmental fluctuations in personality traits were observed, it can be assumed that despite being associated with some aspects of social cognition (with most of the dimensions positively correlating with RFQ or PMS or both), the personality dimensions develop significantly earlier in life and remain relatively stable into adulthood [66].

Finally, the dissimilar pattern between the social cognition variables and the personality dimensions supports the discriminant validity of the RFQ and PMS, partially alleviating the concern that the developmental curve can be explained by the tendency of adolescents to gradually become better at filling in surveys.

### Social cognition and the big five personality traits

To our knowladge the present study was the first to examine the relationship between self-reported mentalizing and the big five personality traints in an adolescent sample. A significant positive relationship was found between mentalizing and psychological mindedness and the personality traits of Agreeableness, Openness to Experience and Conscienciousness.

These findings are consitent with previous research that established a positive correlation between mentalizing [62], psychological mindedness [67] and Agreeableness.

Similary, the connection found in this study between psychological mindedness and Openness to Experience, is also in line with previous research [49] with the present findings extending this assoication to mentalizing. This may be explained by the fact that central traits assoiciated with Openness to Experience are curiosity and a willigness to absorb new ideas—traits also important in mentalizing. It is also suggested that Openness to Experience is related to Conte’s definition of psychological mindedness, or openness to “intrapsychic and interpersonal experience”. A more psychologically minded person tends to be more curious about the behaviors of others and to be more likely to enter into conversations, demonstrating both Extraversion and Openness to Experiences [49].

The findings are in line with the study by Salaminios et al [68] who found that schizotypal personality traits that impede interpersonal contact with others were associated with deficits in mentalizing in an adolescent sample. It is well established that close interpersonal relationships and congurent mirroring of one’s emotions are fundamental in acquisition of mentalizing in early life. However, it is possible that the ability to engage in new experiences and form new close interpersonal relationships in adolescnce may further promote and elaborate mentalizing capacites. At the same time, withdrawing from new experiences and relationships can impede this developent.

From a developmental perspective, it is plausible that young people who are more open to experiences will tend to engage in new interpersonal relationships (or other novel experiences) that can promote the elaboration of their mentalizing skills. Conversely, someone who is less open to experience may restrict their opportunities to use new connections with others to develop their mentalizing skills.

The links between Conscienciousness and both mentalizing and psycholoigcal mindedness found by the present study have not been previously established. It is plausible that they may have to do with the tendancy of highly consciencious people to be thoughtful and aim to understand how their behaviors affect others. Both traits require robust mentalising as they involve both good understanding of one’s own thoughts, feelings and actions, and the imagination to understand the mental states of others and the ways in which one’s behaviors might impact them.

The results of this study show a weaker yet significant association between Extraversion and psychological mindedness; this link was also previously established in the literature [49]. The lack of association with mentalising may be explained by Extraversion being characertised by sociability and talkativeness, both may not necessarily be features of mentalizing, that is more focused on introspectative and implicit processes and less on explicit willingess to share and self-disclose captured by psychological mindedness.

### Social cognition development and gender

The increase in both mentalizing and psychological mindedness observed in males in this study from the age of 14 to 17–18 could be related to the gradual move from mid adolescence to late adolescence. The absence of this change in the female sample could be a result of their faster maturation, suggesting that the initial increase in mentalizing and psychological mindedness in females could occur earlier than the age of 14. As girls reach adolescence earlier and begin engaging socially more than boys [69], this may result in the earlier development of mentalizing and psychological mindedness. This view is also supported by the earlier onset of puberty in girls, which affects frontal lobe development, meaning that a significant gender difference in the development of frontal activity associated with social cognition is to be expected [29]. This earlier development could also account for the significantly lower mentalizing and psychological mindedness in boys at ages 14 to 16. By the age of 17–18 the males seem to catch up and both facets become similar between genders. Further research examining social cognitive development in younger age groups is necessary to support this assumption.

The findings of the current study not only show a strong link between mentalizing and psychological mindedness, but also suggest a temporal difference in the acquisition process of both abilities between the genders. It seems that males improve their capacity to mentalize earlier than their psychological mindedness. Moreover, after the significant increase in the capacity for both mentalizing and psychological mindedness that occurs in the transition to young adulthood, there is no difference observed between genders in psychological mindedness. However, females show significantly higher mentalizing capacity than males at this same stage. These results replicate previous findings, indicating female advantage in mentalizing [14,69,70], suggesting that self-report measures can be sensitive enough to detect these differences. The gender invariance of psychological mindedness in young adulthood can be accounted for by the fact that whilst the conscious cognitive capacities of both genders are equally developed, the more automated, “intuitive” capacities associated with mentalizing are more developed in females [70].

The present findings also seem to indicate that mentalizing may develop earlier than psychological mindedness. As mentioned previously, mentalizing is operating both explicitly and implicitly whilst psychological mindedness is predominantly explicit and conscious. The willingness and ability to *talk* to others about one’s feelings, problems and mental states was also identified as one of the main factors influencing the variance in the PMS scores in both psychiatric and normative samples [60,71]. On careful examination of the self report items it is clear that while both the PMS and the RFQ enquire about the curiosity and willingness of the respondent to explore their own mind—mentalizing the self, the PMS however does not include items enquiring about mentalizing the other. The RFQ on other hand does not capture one’s willingness to talk with others about mental states and receive advice. It is possible that the capacity to mentalize may develop earlier than the ability to more fully verbalize what is on one’s mind and the understanding of the value of sharing ones mental states with others. This assumption is consistent with the significant increase of self-disclosure in adolescence, which is thought to be an important developmental milestone enabling the gradual shift towards peers for social and emotional support [72]. Furthermore, it is possible that the non-conscious, automatic capacities involved in mentalizing may develop earlier than the ability to verbalize and consciously reflect upon one’s own state of mind and that of others. In other words, the adolescent who can intuitively adjust his/her behavior by interpreting the behaviors of others as being influenced by their mental states may not yet be able to describe this process verbally or even become entirely aware of it. These finding could have interesting implications regarding the ability of adolescents to engage with psychotherapy, and a possible need to adjust expectations or demands for reflecting, sharing and self-disclosure in the process. Further research is needed to explore this application.

Finally, an important implication of the difference in the development and gender profiles between mentalizing and psychological mindedness presented in this study, is the notion that even the most similar facets of social cognition seem to develop differently in males and females. Further investigation is needed into these age/gender effects. These finding also invite further investigation into the wider variety of social cognition components and their potentially unique developmental trajectories. Future studies should consider the prolonged nature of adolescence in modern society [5], as supported by neurological studies [73], which effectively erases the clear distinction between late adolescence and young adulthood.

### Limitations and future research directions

There were several limitations to the current study. Firstly, the tool used to measure mentalizing (RFQ) had the limitations of being a self-report measure. It is likely that the measure did not capture the full complexity and subtlety of the concept of mentalizing. The same can be said about the PMS as a measure of psychological mindedness. Furthermore, the standard limitations of self-reporting such as biased perception, social desirability, false reporting, etc. [74], should be taken into account when interpreting the findings. However, in the above weakness lies a potential strength, as the trends that were found in the present study are highly consistent with both experimental data and neuroscientific evidence. The consistency suggests that self-report can be used as a relevant proxy to measure social cognition. Most studies to date exploring the developmental features of social cognition in adolescence utilized neuroimaging, ToM tasks or interviews, making it very labour-intensive to measure. Self-report measures may be less accurate but are a significantly more efficient tool. However, future studies utilizing mixed methodologies are necessary to substantiate this claim.

A recent study has suggested that the original scoring of the RFQ [58] should be used with caution as recent findings suggest that the measure is more appropriate as a unidimensional one rather than capturing two dimensions of certainty and uncertainty [75]. While the present study addressed this criticism and used a unidimensional coding version of the RFQ, further research is required to establish which of the coding systems is the most suitable to measure mentalizing. It is likely that different scoring is appropriate for clinical and non-clinical samples.

A further group of limitations is associated with the cross-sectional design of the study, which could cause some of the findings to be confounded by a third variable. Furthermore, the inability to establish temporal ordering among variables introduces the possibility of reverse-causation. Moreover, the inability to account for cohort effects could bias the suggested estimates of developmental change. To address these and provide a more comprehensive picture of the developmental trajectory of social cognition through adolescence and early adulthood future studies should adopt a longitudinal design.

Other limitations of the study were related to the relative homogeneity of the sample used and the limited demographic data collected. These invite caution in inferring the generalisability of the findings. This sample consisted of participants of a relatively high socio-economic background and higher than average cognitive abilities due to the selective nature of the two independent schools and the universities. Furthermore, the study did not account for past or present psychiatric or neurogenetic conditions that could affect the participants’ mentalizing. It is important to replicate these findings in a more diverse population, as some of the reported developments could be associated with less adversity experienced in the sample—a factor known to influence the development of social cognition [76]. Similarly, the sample was culturally and linguistically homogenous, with the majority of participants indicating English as their first language. Future research could examine whether cultural differences can be observed in the development of social cognition.

Furthermore, the 14 to 18 years sample was recruited from single sex schools where the sample had less exposure to the opposite sex. This could have influenced the development of social cognition. Future studies should also examine these developmental trends in co-educational settings.

Finally, the study showed a significant change in both mentalizing and psychological mindedness at some point between the ages of 18 to 20. A larger sample of each age group, with smaller age range bands, may allow for more detailed understanding of this gradual development.

## Conclusion

The current self-report study has attempted to measure two components of social cognition—mentalizing and psychological mindedness—never previously measured alongside each other in adolescence. It provides preliminary evidence for the similarity in the development of these two facets of social cognition through adolescence and into adulthood. Important differences in their acquisition associated with age and gender differences were also found and previously unreported associations between the two concepts and some of the Big Five Personality Traits were established. The findings of the study were in line with current research on social cognition and brain development and generally reflected the developmental tendencies in adolescence. They suggest that gender, age and personality traits should all be considered in order to establish a fully integrative picture of social-cognitive development in adolescence. This study also provides further evidence that self-report can be used to measure constructs associated with social cognition in adolescence, making such research less dependent upon resource heavy measures such as neuroimaging or complicated experimental designs. Overall, this study points to developmental changes in mentalizing and psychological mindedness capacities, which peaks during the transition from late adolescents into young adulthood [1].
